# *CDKN2A*-Mutated Pancreatic Ductal Organoids from Induced Pluripotent Stem Cells to Model a Cancer Predisposition Syndrome

**DOI:** 10.3390/cancers13205139

**Published:** 2021-10-13

**Authors:** Jessica Merkle, Markus Breunig, Maximilian Schmid, Chantal Allgöwer, Jana Krüger, Michael K. Melzer, Susanne Bens, Reiner Siebert, Lukas Perkhofer, Ninel Azoitei, Thomas Seufferlein, Sandra Heller, Matthias Meier, Martin Müller, Alexander Kleger, Meike Hohwieler

**Affiliations:** 1 Department of Internal Medicine I, Ulm University Hospital, 89081 Ulm, Germany; Jessica.Merkle@uni-ulm.de (J.M.); Markus.Breunig@uni-ulm.de (M.B.); max-schmid92@web.de (M.S.); chantal.allgoewer@uni-ulm.de (C.A.); jana.krueger@uni-ulm.de (J.K.); Michael.Melzer@uniklinik-ulm.de (M.K.M.); lukas.perkhofer@uniklinik-ulm.de (L.P.); ninel.azoitei@uni-ulm.de (N.A.); thomas.seufferlein@uniklinik-ulm.de (T.S.); sandra.heller@uni-ulm.de (S.H.); martin.mueller@uniklinik-ulm.de (M.M.); 2 Department of Urology, Ulm University, 89081 Ulm, Germany; 3 Department of Human Genetics, Ulm University & Ulm University Hospital, 89081 Ulm, Germany; susanne.bens@uni-ulm.de (S.B.); reiner.siebert@uni-ulm.de (R.S.); 4 Helmholtz Pioneer Campus, Helmholtz Zentrum München, 85764 Neuherberg, Germany; matthias.meier@helmholtz-muenchen.de

**Keywords:** familial pancreatic cancer, induced pluripotent stem cells, pancreatic organoids, CDKN2A

## Abstract

**Simple Summary:**

The mutational landscape of pancreatic ductal adenocarcinoma has been delineated in large clinical studies. Such retrospective characterization, however, is limited to late stages of the disease, and exploring the complexity of carcinogenic routes is in its infancy. Here, we provide an in vitro and in vivo model system to directly investigate human PDAC development after the induction of specific oncogenic insults in a cancer-prone ancestry. We reprogrammed plucked human hair-derived keratinocytes from two siblings harboring a pathogenic *CDKN2A* variant putatively predisposing for PDAC to induced pluripotent stem cells (iPSCs) and additionally introduced an inducible *KRAS^G12D^* *piggyBac* expression cassette. Upon *KRAS^G12D^* induction in differentiated pancreatic duct-like organoids, structural and molecular changes were observed. After orthotopic transplantation either a high-grade precancer lesion or PDAC-like tumor developed. Hereby, we provide a hereditary human pancreatic cancer model which enables further dissection of tumor initiation and early development starting from patient-specific *CDKN2A*-mutated pluripotent stem cells.

**Abstract:**

Patient-derived induced pluripotent stem cells (iPSCs) provide a unique platform to study hereditary disorders and predisposition syndromes by resembling germline mutations of affected individuals and by their potential to differentiate into nearly every cell type of the human body. We employed plucked human hair from two siblings with a family history of cancer carrying a pathogenic *CDKN2A* variant, P16-p.G101W/P14-p.R115L, to generate patient-specific iPSCs in a cancer-prone ancestry for downstream analytics. The differentiation capacity to pancreatic progenitors and to pancreatic duct-like organoids (PDLOs) according to a recently developed protocol remained unaffected. Upon inducible expression of *KRAS^G12D^* using a *piggyBac* transposon system in CDKN2A-mutated PDLOs, we revealed structural and molecular changes in vitro, including disturbed polarity and epithelial-to-mesenchymal (EMT) transition.* CDKN2A*-mutated *KRAS^G12D^* PDLO xenotransplants formed either a high-grade precancer lesion or a partially dedifferentiated PDAC-like tumor. Intriguingly, P14/P53/P21 and P16/RB cell-cycle checkpoint controls have been only partly overcome in these grafts, thereby still restricting the tumorous growth. Hereby, we provide a model for hereditary human pancreatic cancer that enables dissection of tumor initiation and early development starting from patient-specific* CDKN2A*-mutated pluripotent stem cells.

## 1. Introduction

Pancreatic ductal adenocarcinoma (PDAC) is one of the most devastating diseases of the exocrine pancreas and ranks 7th among cancer-related deaths worldwide with a 5-year survival rate of only 9% [1]. Up to 10% of PDACs occur in families with at least two affected first-degree relatives. Whole genome sequencing of such familial pancreatic cancer (FPC) patients has recently elucidated the heterogeneous mutational landscape and outlined the importance of mutations in susceptibility genes like *ATM*, *BRCA2*, and *CDKN2A* (cyclin-dependent kinase inhibitor 2A) [2,3,4,5]. In fact, mutations in the latter appear to be the third most prevalent in FPC while at the same time loss of* CDKN2A* is one of the key oncogenic events in sporadic PDAC [2,6,7]. Furthermore, *CDKN2A* is affected in up to 40% of hereditary melanoma families [8,9] and is associated with familial atypical multiple mole melanoma (FAMMM) syndrome, which is characterized by a family history for melanoma and the occurrence of multiple melanocytic nevi [10]. Importantly, the risk of developing pancreatic cancer is 13- to 22-fold increased for individuals suffering from the FAMMM syndrome [11].

Human pluripotent stem cells (hPSCs) provide a unique platform for developmental and biomedical studies due to their capability to differentiate into many cell types of the human body [12]. Moreover, the use of patient-derived induced pluripotent stem cells (iPSCs) allows disease modeling in a patient-specific background. Our recently developed differentiation protocol enables the generation of pancreatic duct-like organoids (PDLOs) from pluripotent stem cells carrying specific cancer-associated mutations [13,14]. In contrast to end-stage analysis in tumor organoids [15], such an hPSC-based model system offers the potential of in vitro investigation of cancer evolution in a stage-specific and timed manner [13,16,17]. In addition, orthotopic xenotransplantation allows the generation of human PDAC-like tumors in mice for disease modeling in vivo [13,17,18].

Here, reprogramming of *CDKN2A*-mutated patient cells was combined with genomic engineering using a *piggyBac* transposon system [19] to enable timed expression of the *KRAS^G12D^* oncogene in hPSC-derived PDLOs in vitro as well as in vivo.

## 2. Results

### 2.1. Generation of Patient-Specific CDKN2A-Mutated Induced Pluripotent Stem Cells

**Figure 1:**
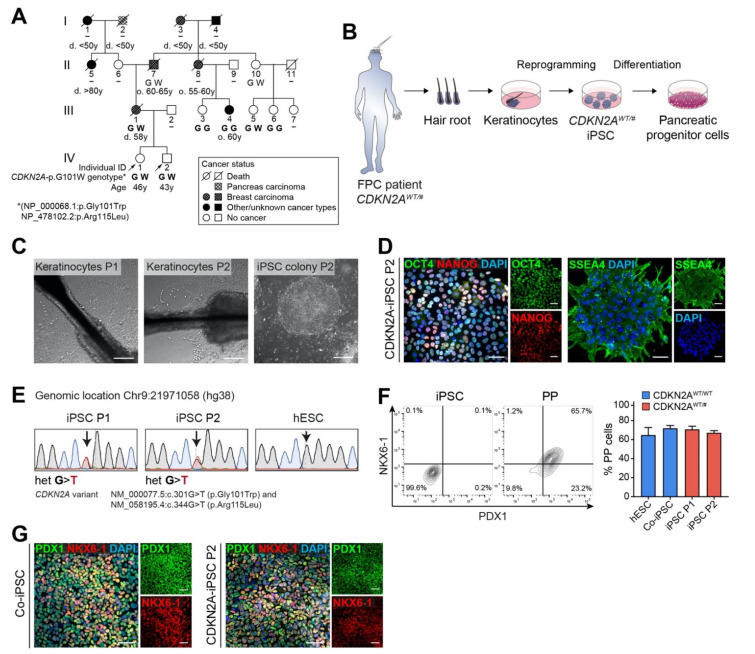
(**A**) Pedigree of a pair of siblings carrying a heterozygous *CDKN2A* germline variant concurrently resulting in known missense variants of the two transcripts *P16* and *P14*, NM_000077.4:c.301G>T (NP_000068.1:p.Gly101Trp) and NM_058195.3:c.344G>T (NP_478102.2:p.Arg115Leu). For clarity, this genotype will be hereafter referred to as *CDKN2A*^WT/#^. In the diagram, only the amino acid change in P16 (G101W) is indicated for each relative, if known. Age at death is indicated by a d., age of onset of the disease by an o. Individual II-5 developed uterine cancer at a young age and bladder cancer at an older age. III-4 suffers from a carcinoma of the external auditory canal, whereas the cancer entities of I-1 and I-4 were unknown. Consultant probands P1 (IV-1) and P2 (IV-2) are highlighted with an arrow. If the *CDKN2A* variant was confirmed by genetic analysis, it is displayed in bold. The displayed age denotes the age at which cancer was diagnosed. (**B**) Schema of iPSC generation by reprogramming of *CDKN2A*^WT/#^ patient hair keratinocytes and subsequent differentiation to pancreatic progenitors (PPs). (**C**) Keratinocytes growing out from a hair root and an iPSC colony derived from reprogrammed keratinocytes. (**D**) IF staining confirmed expression of pluripotency markers OCT4, NANOG, and SSEA4 in the generated patient-specific iPSCs. Scale bar: 50 µm. (**E**) Sequencing of iPSCs from both donors confirming the heterozygous *CDKN2A* variant, chr9:g.21971058C>A (hg38). (**F**) Patient-specific iPSCs differentiated efficiently into PPs. Representative original flow cytometry plots of undifferentiated iPSCs and *CDKN2A*^WT/#^ P2 iPSCs differentiated for 13 days to PPs are shown next to quantification of PDX1/NKX6-1 double positive cells. *n* = 3; ordinary one-way ANOVA with Tukey’s multiple comparison test. (**G**) IF staining for PP marker PDX1 and NKX6-1. Scale bar: 50 µm.

To provide a model for a putative human pancreatic cancer predisposition syndrome, we generated iPSCs from two siblings with a family history of cancer, including several mammary gland tumors and one pancreatic ductal adenocarcinoma (PDAC) (Figure 1A). A heterozygous pathogenic variant in the germline in the *CDKN2A* locus, chr9:g.21971058C>A (hg38), has been previously identified through targeted panel-sequencing of the mother of the subjects investigated in this study (individual III-1). She suffered from triple-negative breast cancer, and external panel sequencing of 94 genes that are associated with hereditary tumor disease detected a genetic pathogenic aberration in *CDKN2A*. The specific pathogenic variant leading to mutant P16 (p.G101W) and P14 (p.R115L), the two main proteins encoded by *CDKN2A*, was transmitted to both siblings (Figure 1A). Pathogenic point mutations and deletions in the BRCA1/2 gene have been found neither in preserved blood of the deceased mother (III-1) nor in the consulting daughter (IV-1). For modeling FPC, plucked hair of the two siblings (P1, IV-1, and P2, IV-2) was obtained to isolate keratinocytes growing out of the outer root sheath (Figure 1B,C). Reprogramming factors were introduced via lentiviral infection with hOKSM-dTomato encoding OCT4, KLF4, SOX2, and MYC. Transduced cells successfully formed iPSC colonies cultured on a feeder layer of rat embryonic fibroblasts [20] (Figure 1C). Immunofluorescence (IF) staining validated pluripotency of the generated iPSCs illustrating the expression of the stem cell markers OCT4, NANOG, and SSEA4 (Figure 1D). DNA from selected clonal iPSC lines was analyzed by Sanger sequencing of exon 2 of the *CDKN2A* gene, which confirmed the heterozygous pathogenic *P16/P14* variant (NM_000077.5:c.301G>T/NM_058195.4:c.344G>T, hereafter denoted *CDKN2A^WT/#^*) in iPSCs from both patients (Figure 1E).

A recent study showed that knockdown of *CDKN2A*-encoded proteins in early pancreatic endoderm cells resulted in higher numbers of pancreatic progenitor cells (PPs) in an in vitro hESC differentiation approach [21]. Therefore, we first aimed to elucidate a possible impact of the described *CDKN2A* germline variant on pancreatic commitment of patient-derived iPSCs. We differentiated the generated cell lines to PPs through our recently established 13-day protocol [13] and evaluated the amount of PPs characterized by co-expression of the transcription factors PDX1 and NKX6-1. However, flow cytometry revealed efficient PP generation from P1- and P2-iPSC lines with similar numbers of PDX1/NKX6-1 positive cells as PPs derived from control iPSCs (Co-iPSCs) and human embryonic stem cells (hESC, HUES8) (Figure 1F). Additional IF staining confirmed the homogenous induction of PDX1/NKX6-1 from *CDKN2A*-mutated iPSCs (Figure 1G).

### 2.2. CDKN2A-Mutated PDLOs Display Oncogenic Effects after KRAS^G12D^ Induction

**Figure 2:**
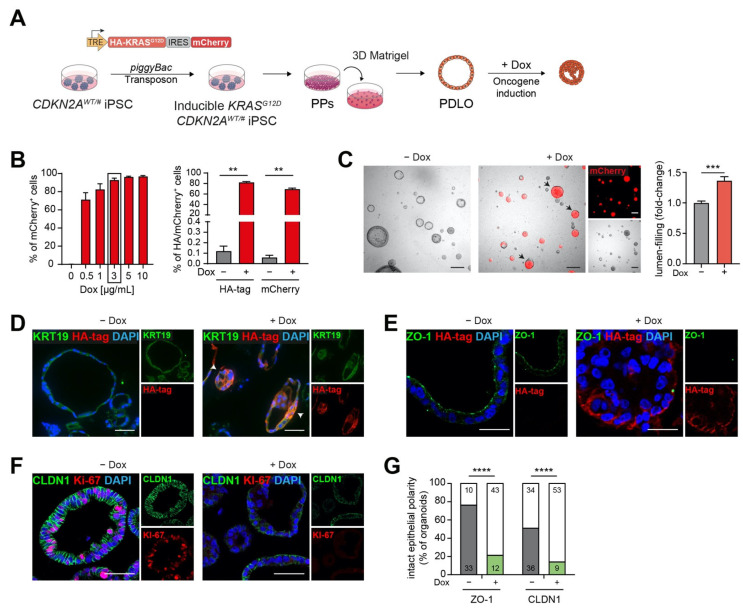
(**A**) Schema of modeling FPC in vitro. Integration of the inducible *KRAS^G12D^ piggyBac* construct in *CDKN2A*^WT/#^ patient iPSCs followed by directed differentiation to pancreatic duct-like organoids (PDLO) and oncogene activation by doxycycline (Dox) treatment. (**B**) Left: quantification of mCherry reporter expression in d27 PDLOs established from **KRAS^G12D^* CDKN2A^WT/#^* iPSCs analyzed by flow cytometry after treatment with different Dox concentrations for 9 days; *n* = 2; in duplicates. Right: Flow cytometry analysis of transgenic *KRAS^G12D^* expression by HA-tag staining or direct measurement of the mCherry reporter signal; *n* = 3; in duplicates, Mann-Whitney test. (**C**) Left: bright field images of *CDKN2A*-mutated iPSC-derived PDLO cultures with or without oncogene induction for 9 days. Arrowheads highlight several lumen-filled PDLO structures. Scale bar: 200 µm. Right: the number of lumen-filled organoids was significantly increased after oncogene induction; *n* = 3; in triplicates, Mann-Whitney test. (**D**) IF staining of the HA-tag confirms successful transgene expression of *KRAS^G12D^* after Dox stimulation in PDLOs in vitro. Arrowheads point to disorganized PDLO structures. (**E**) Loss of apical ZO-1 in *KRAS^G12D^*-expressing *CDKN2A*^WT/#^ patient PDLOs detected by IF staining. Scale bar: 25 µm. (**F**) IF staining of Ki-67 and tight junction protein CLDN1 indicate reduced proliferation and disrupted polarization, respectively. (**G**) Organoids were quantified in a blinded manner. Structures with a clear luminal ZO-1 expression, or with at least 50% of cells showing a strong basolateral CLDN1 localization, were counted as positive organoids. PDLOs lost epithelial polarity upon *KRAS^G12D^* induction (ZO-1: Odds ratio 11.83 with 95% confidence interval 4.56–30.70; CLDN1: Odds ratio 6.24 with 95% confidence interval 2.67–14.56); *n* = number of organoids, IF images from two cell lines were quantified; Fisher’s exact test. CLDN1: Claudin-1; ZO-1: Zona occludens-1. Scale bar: 50 µm, if not stated differently. Experiments were performed as end point analysis 7 or 9 days after treatment with 3 µg/mL Dox. (**B**,**C**,**G**), ** *p* < 0.01, *** *p* < 0.001, **** *p* < 0.0001.

*KRAS* mutations are a hallmark of PDAC and represent the most common second oncogenic hit in cases of FPC with predisposing germline mutations [22]. To mimic the occurrence of such additional somatic mutations in patients during their lifetime, *CDKN2A*^WT/#^ iPSCs from P1 were further engineered with a Tet-On cassette allowing conditional expression of *KRAS^G12D^*/mCherry using an “All-in-one” *piggyBac* transposon system [19]. This construct encodes for *KRAS^G12D^*, the constitutively active form of the GTPase KRAS, which was tagged with an N-terminal HA sequence and linked to a mCherry reporter facilitating transgene detection (Figure 2A).

Equipped with this construct, *KRAS^G12D^* *CDKN2A^WT/#^* iPSCs were differentiated into PDLOs following our novel differentiation platform to efficiently direct human PSCs towards the ductal pancreatic lineage in a Matrigel-based 3D culture [13] (Figure 2A). Doxycycline (Dox) was added to the cultures, and mCherry reporter and HA-tag expression confirmed a robust transgene induction in a dose-dependent manner (Figure 2B). *KRAS^G12D^* induction caused the formation of compact lumen-filled organoid structures (Figure 2C). Interestingly, this spherical organoid morphology was previously shown to be characteristic for KRAS mutant organoids growing from PDAC specimens in contrast to normal cystic organoids, suggesting that our system recapitulates oncogenic KRAS-driven transformation [13,23]. IF staining of KRT19 confirmed ductal identity of **KRAS^G12D^* CDKN2A^WT/#^* iPSC-derived organoids, and expression of HA-tagged *KRAS^G12D^* correlated with less organized PDLO structures (Figure 2D). Moreover, the changes in tissue architecture upon *KRAS^G12D^* induction correlated with a disruption of apico-basal polarity. Expression of the tight junction protein ZO-1 was significantly lost (Figure 2E,G) and basolateral expression of CLDN1 in untreated PDLOs shifted to weaker and less distinct membranous expression in Dox-induced PDLOs (Figure 2F,G).

**Figure 3:**
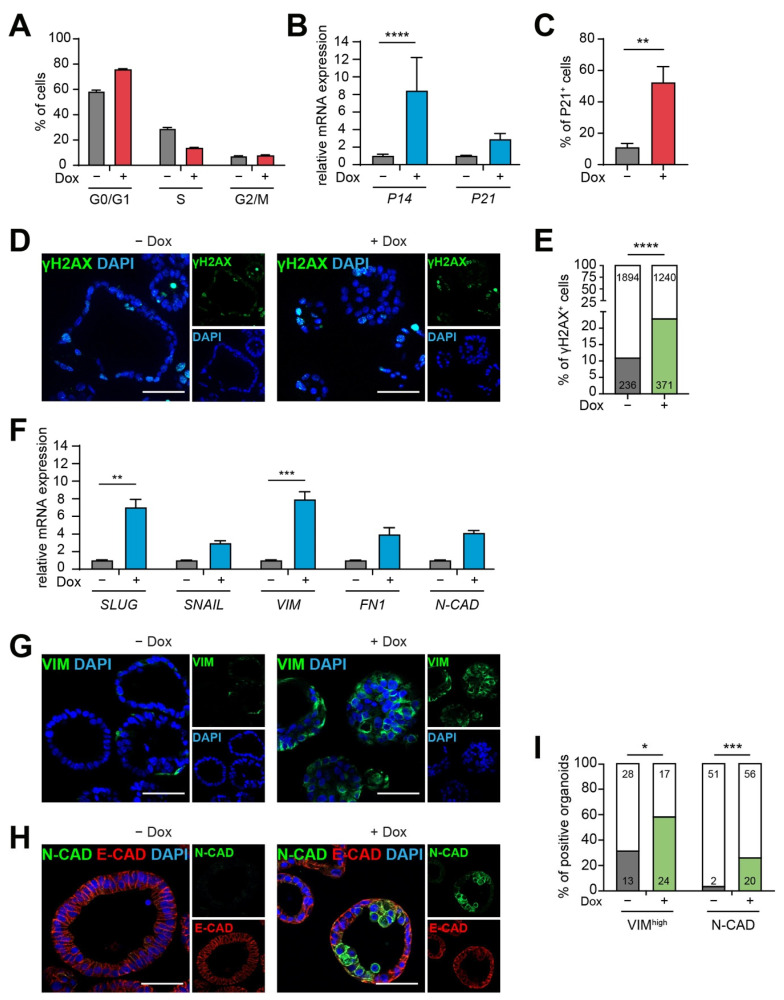
(**A**) Flow cytometry-based cell cycle analysis by EdU staining shows a tendency to reduced S-phase and a G0/G1 arrest after 7 days of Dox-induced *KRAS^G12D^* overexpression; *n* = 2; in duplicates. (**B**,**C**) Analysis of cell cycle regulators confirmed (**B**) transcriptional upregulation of *P14* by qPCR and (**C**) an increased number of P21 positive cells measured by flow cytometry. (**D**) IF images and (**E**) respective semi-automated quantification of the DNA damage marker γH2AX. More cells were γH2AX positive upon *KRAS^G12D^* induction (Odds ratio 2.40 with 95% confidence interval 2.01–2.87). (**F**) qPCR analysis for EMT transcription factors (*SLUG, SNAIL*) and mesenchymal markers (*VIM, FN1, N-CAD*) shows the upregulation of an EMT program upon KRAS induction. (**G**–**I**) Increased expression of mesenchymal proteins in Dox-treated organoids. IF staining for (**G**) VIM and (**H**) N-CAD/E-CAD and (**I**) respective image-based quantification. Organoids were quantified in a blinded manner. Organoids were counted as VIM^high^ when a strong VIM signal was seen in PDLOs, whereas organoids were counted as N-CAD positive when at least one cell within an organoid was N-CAD positive. PDLOs acquired mesenchymal features upon *KRAS^G12D^* induction (VIM: *P*-value: 0.0259, Odds ratio 3.04 with 95% confidence interval 1.23–7.52; N-CAD: P-value: 0.0007; Odds ratio 9.11 with 95% confidence interval 2.03–40.91). (**B**,**C**,**F**): end point analysis after 7 or 9 days Dox treatment; *n* = 3; in duplicates, (**B**,**F**) two-way ANOVA with Sidak’s multiple comparison test, (**C**) Mann-Whitney test. (**E**,**I**): Dox treatment for 7 days; *n* = number of organoids, IF images from two cell lines were quantified; Fisher’s exact test. * *p* < 0.05, ** *p* < 0.01, *** *p* < 0.001, **** *p* < 0.0001. In all experiments, Dox was applied at a concentration of 3 µg/mL. E-CAD: E-cadherin; FN1: Fibronectin-1; N-CAD: N-cadherin; VIM: Vimentin. Scale bars: 50 µm.

We also observed reduced Ki-67 immunoreactivity in cells of KRAS-expressing PDLOs (Figure 2F) and thus further investigated the proliferation by EdU-assay. Indeed, we found a trend towards a higher fraction of cells in G0/G1 phase of the cell-cycle and, in turn, less cells in the S-phase (Figure 3A). In agreement, the expression of cell cycle inhibitors *P14* and *P21* was upregulated (Figure 3B,C). P14, which is encoded by the *CDKN2A* locus, can stabilize P53 by inhibiting MDM2-mediated degradation, and P21 is then activated downstream of P53 [24]. In addition to persistent KRAS signaling, P14 and P21 may be upregulated as response to DNA damage. We therefore analyzed the phosphorylation of histone H2AX (γH2AX) and detected a significant increase of γH2AX-positive cells upon Dox treatment (Figure 3D,E) indicating replication stress and DNA damage in *KRAS^G12D^* overexpressing *CDKN2A^WT/#^* PDLOs.

Loss of epithelial polarity as well as decreased proliferation can be associated with epithelial-to-mesenchymal transition (EMT) [25,26,27]. We recently described the initiation of an EMT-like program through oncogenic KRAS in hESC-derived PDLOs [13]. Hence, we performed qPCR analysis and, indeed, *KRAS^G12D^* induced the expression of various EMT marker genes in the patient iPSC-derived PDLOs (Figure 3F). In addition, a significant increase of Vimentin (VIM) and N-cadherin (N-CAD) protein expression confirmed the gain of a mesenchymal phenotype (Figure 3G–I). Hence, *CDKN2A^WT/#^* PDLOs did not only undergo structural changes but also acquired certain molecular EMT properties within 7–9 days of *KRAS^G12D^* induction.

### 2.3. Tumor Formation in Xenotransplantation Experiments

**Figure 4:**
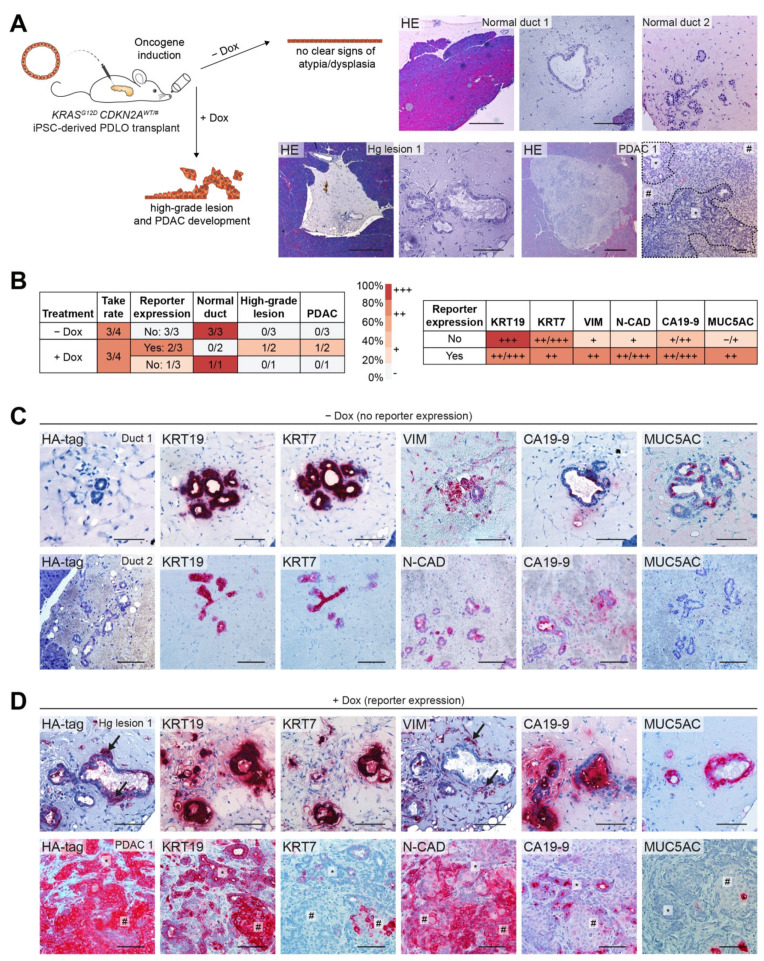
(**A**) Schema of modeling FPC in vivo. *KRAS^G12D^* was induced for 8 weeks after orthotopic transplantation of *CDKN2A^WT/#^* iPSC-derived PDLOs in NSG mice. HE staining of identified engraftments with and without Dox treatment. Scale bar: 500 µm (overview), 100 µm (magnification); Hg lesion: high-grade lesion. The asterisk marks a glandular region, the pound sign a highly atypical and dysplastic region of the invasive PDAC-like tumor graft. (**B**) Summary of engraftment experiments. Left: Take rate was determined by identified engraftments per transplanted mice. Reporter expression was analyzed by mCherry and HA-tag IHC staining, and grafts were classified into normal ducts, low-grade lesions (non found), high-grade lesions, and PDAC-like tumors by the observed degree of atypia, dysplasia, and invasiveness. Right: Semi-quantitative marker expression: Each engraftment was classified into no (–), weak (+), moderate (++), or strong (+++) expression. The mean expression across the engraftments of one group is depicted. (**C**) *CDKN2A^WT/#^* PDLO grafts without oncogene induction formed epithelial duct-like tissue (KRT19, KRT7). Some EMT marker expression (VIM, N-CAD) was accompanied by moderate expression of CA19-9. MUC5AC was only expressed in one graft. (**D**) Two *CDKN2A^WT/#^* PDLO grafts 8 weeks after oncogene induction. The heterogeneous HA-tag expression in Hg lesion 1 correlated with increased dissemination and EMT, e.g., indicated by HA-tag and VIM staining (regions marked by arrows). This graft also strongly upregulated MUC5AC and CA19-9. The second invasive tumor graft (PDAC 1) with high levels of HA-tag expression was partially dedifferentiated (KRT19, KRT7, N-CAD) and only expressed CA19-9 and MUC5AC in some regions. All scale bars: 100 µm, if not stated differently.

To investigate the tumor formation capacity of *CDKN2A*^WT/#^ iPSC-derived pancreatic progeny *per se* and together with an additional oncogenic hit, PDLOs harboring an inducible *KRAS^G12D^* transgene were orthotopically transplanted into immunocompromised NSG mice. Oncogene expression was induced in one group of mice by the addition of Dox to the drinking water for 8 weeks (Figure 4A). Whereas patient-derived *CDKN2A*^WT/#^ PDLOs formed morphologically normal one-layered duct-like tissue in vivo, engraftments with detected reporter expression, indicative for *KRAS^G12D^* induction, either resembled a high-grade precancer lesion or an invasive PDAC-like tumor (Figure 4A,B left). In the first *CDKN2A*^WT/#^ *KRAS^G12D^* engraftment, signs of cellular atypia, such as the increased size of nuclei and cytoplasm and the disorganization of epithelial structures, were accompanied by dysplastic growth into the surrounding tissue (Figure 4A). Few cells appeared to invade the basal membrane, resembling a high-grade preneoplastic lesion with early signs of cancerous growth. The second engraftment developed into a palpable tumor with a glandular compartment (asterisk) and a highly atypical and dysplastic compartment (pound sign) (Figure 4A). Strong KRT19 and KRT7 expression in *CDKN2A*^WT/#^ engraftments confirmed the ductal identity of the derived tissue (Figure 4B right,C), albeit some cells expressed VIM and N-CAD without Dox treatment (Figure 4B right,C). Conclusively, these grafts expressed low to moderate levels of CA19-9, while one graft expressed MUC5AC. CA19-9 can be either a sign of further ductal maturity or dysplasia, but MUC5AC is usually not found in untransformed normal ducts.

In comparison to *CDKN2A*^WT/#^ engraftments, *KRAS^G12D^* induction led to a reduction in epithelial markers KRT19 and KRT7 and in turn an increase in mesenchymal markers VIM and N-CAD, as well as dysplasia markers CA19-9 and MUC5AC (Figure 4B right, D). VIM positive cells in the high-grade lesion were particularly observed in regions of dissemination from the ductal epithelium (indicated by arrows) and correlated with strong HA-tag expression (Figure 4D). Similarly, regions with strong HA-tag expression in the PDAC tumor graft also expressed less KRT19 and KRT7 and substantially higher levels of mesenchymal markers such as N-CAD (Figure 4D, pound sign).

### 2.4. Checkpoint Integrity in CDKN2A^WT/#^ PDLO Grafts

**Figure 5:**
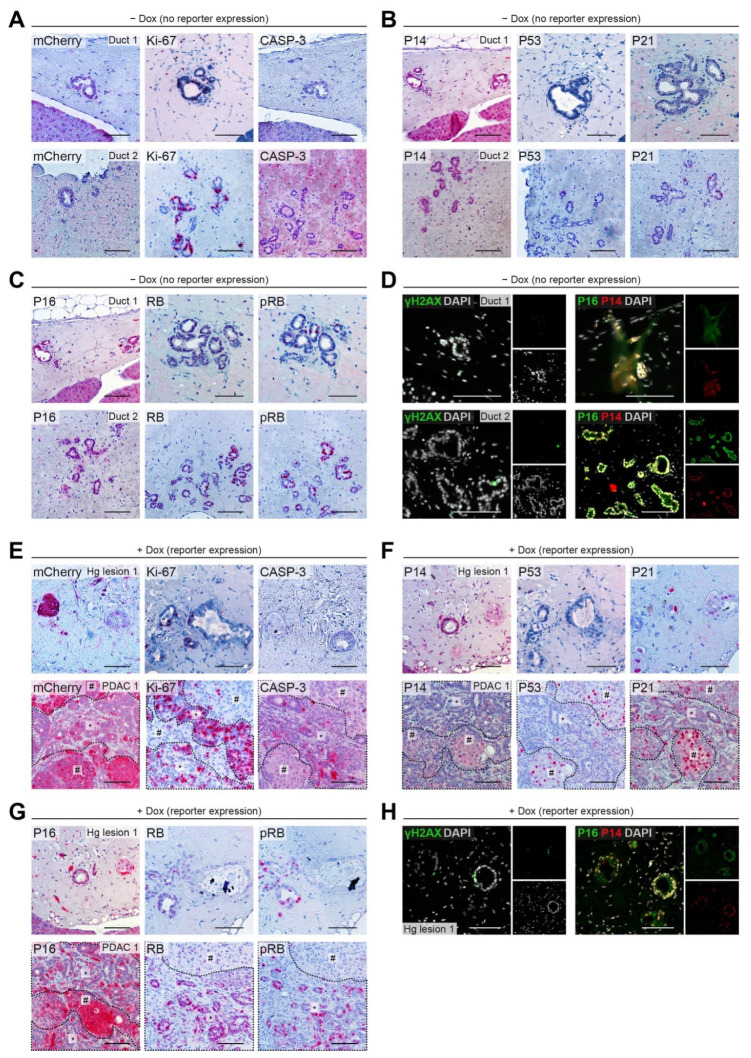
(**A**–**D**) Analysis of cell cycle checkpoint control of *CDKN2A^WT/#^* PDLO grafts. IHC staining for (**A**) reporter expression (mCherry), proliferation (Ki-67), and apoptosis (cleaved CASP-3), (**B**) underlying P14, P53, P21, and (**C**) P16, RB signaling. (**D**) IF analysis of γH2AX and P16/P14. (**E**–**H**) Analysis of cell cycle checkpoint control of *CDKN2A^WT/#^* PDLO grafts after 8 weeks of *KRAS^G12D^* induction analogous to A-D. All scale bars: 100 µm. CASP-3: cleaved caspase 3; (p)RB: (phosphorylated) Retinoblastoma protein.

To further delineate the role of the predisposing *CDKN2A* variant on its tumor suppressive function, we characterized cell cycle progression and proliferation of the arising grafts upon orthotopic transplantation. Although we observed moderate proliferation (Ki-67) in *CDKN2A^WT/#^* PDLO grafts, barely any apoptotic events were detected (cleaved CASP-3) (Figure 5A). Moderate expression of P16 and P14 evoked neither a downstream activation of P53 and P21 (Figure 5B) nor a clear reduction of pRB levels (Figure 5C). In agreement with low levels of cellular stress, indicated by low levels of γH2AX (Figure 5D), the lack of senescence effectors allowed cell cycle progression in *CDKN2A^WT/#^* grafts.

**Figure 6:**
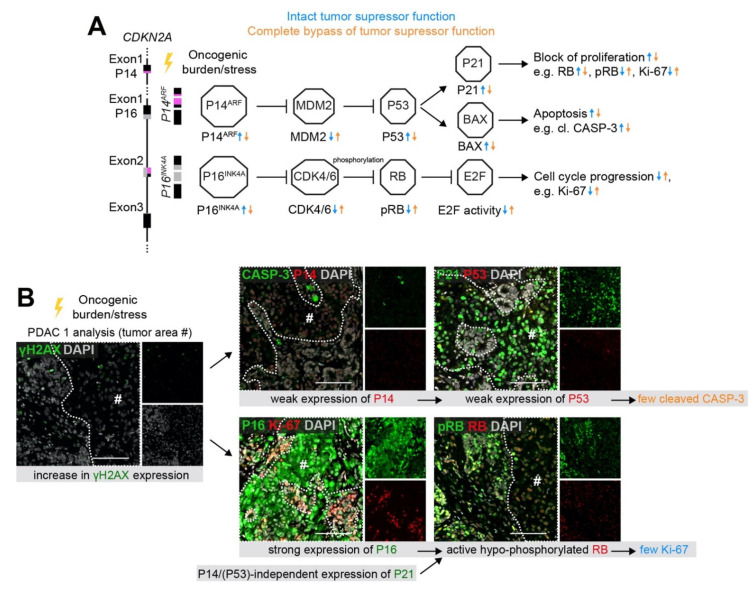
(**A**) Schematic overview of tumor suppressive role of *CDKN2A*. *CDKN2A* encodes for two different transcripts P16 and P14, which are both involved in controlling and restricting cell cycle progression. While the activation of P14 leads to an increase in P53 and P21, the induction of P16 results in an activation of RB by inhibiting its hyper-phosphorylation. Non-canonical, P53- and RB-independent functions of P14 and P16 are not illustrated in this simplified scheme. (**B**) Partial growth restriction in the PDAC-like tumor graft of transplanted *CDKN2A^WT/#^ KRAS^G12D^* PDLOs. In the less proliferative, highly dysplastic tumor regions indicated by a pound sign, P21 was strongly upregulated, probably mainly through a P14/P53 independent mechanism, P16 was highly expressed, and RB was not hyper-phosphorylated indicating an intact cell cycle progression barrier. All scale bars: 100 µm.

In the *CDKN2A^WT/#^* *KRAS^G12D^* PDAC-like tumor, however, we observed that proliferation in highly dysplastic regions (pound sign) was lower than in glandular structures of the same graft (Figure 5E). In contrast, cleaved CASP-3, indicative for apoptosis, was rather seen in glandular structures and not in the highly dysplastic compartment (Figure 5E). The regions marked by low proliferation strongly expressed P21 and P16 resulting in hypo-phosphorylation of RB and growth restriction (Figure 5E–G). The growth restriction following *KRAS^G12D^* induction was also evident in the high-grade precancer lesions by an increase in P21 and relatively low Ki-67 levels (Figure 5E,F). At the same time, γH2AX levels were still rather low to moderate in this lesion (Figure 5H). A schematic overview in Figure 6A illustrates the mode of *CDKN2A*-mediated tumor suppressor function via either P14/P53/P21 or P16/RB. Co-immunofluorescence staining of the *CDKN2A^WT/#^ KRAS^G12D^* PDAC-like tumor further demonstrated that cell cycle roadblocks remained mostly functional (Figure 6B). Intact cell cycle control was demonstrated by hypo-phosphorylation of RB and low levels of Ki-67, both correlating spatially with high levels of P16 and P21 (pound sign in Figure 6B). In contrast, P14 and P53 appeared rather weak with P53 being only detectable in IHC (Figure 5F) but not in IF (Figure 6B), which might translate to low levels of the apoptotic marker cleaved CASP-3 and a partial bypass of tumor barriers.

## 3. Discussion

*CDKN2A*-encoded proteins display essential roadblocks to cancer initiation, and *CDKN2A* ranks high within FPC susceptibility genes [2,7]. We exploited reprogramming strategies in combination with a *piggyBac* engineering approach introducing oncogenic *KRAS^G12D^* to create a human in vitro model system for a pancreatic cancer predisposition syndrome. The use of iPSCs from patients with a family history of cancer allowed us to mimic the potential risk associated with an inherited monoallelic variant of *CDKN2A*. The investigated chr9:g.21971058C>A (hg38) founder mutation is the most frequently observed missense *CDKN2A* variant in familial melanoma cancers [28,29,30] with hints that the specific mutation might confer a higher risk of developing pancreatic cancer than other pathogenic *CDKN2A* variants [31]. However, such association needs further investigations but might be linked to variant-specific alterations of both the P16 and P14 barrier [32,33]. The chr9:g.21971058C>A (hg38) variant likely results in global conformational changes of both proteins, P16 and P14 [34]. Direct head-to-head comparisons of different *CDKN2A* variants will help to elucidate this question.

The impact of a P16-p.G101W/P14-p.R115L germline variant on embryonic pancreas development was first assessed by the implementation of a recently established pancreatic ductal differentiation model [13]. The investigation of developmental stages is of particular relevance since certain rare inherited pancreatic disorders manifest prenatally, as demonstrated in several studies of monogenetic diabetes [35,36,37]. Studies in mice have indeed demonstrated that mutant CDK4, which is insensitive to inhibition by p16^Ink4a^ (*Cdkn2a*), may cause increased proliferation of Pdx1+ pancreatic epithelial cells and expansion of the β-cell mass during embryogenesis [38]. However, we now show that *CDKN2A*^WT/#^ iPSCs from two siblings differentiate with similar high efficiency to PPs as control iPSCs and hESCs, indicating no effect of the *CDKN2A* missense variant on early pancreatic development. *CDKN2A*^WT/#^-derived PPs also efficiently developed into PDLOs and duct-like tissue upon orthotopic transplantation. Albeit arising grafts resembled the morphology of wildtype grafts [13], we observed some degree of mesenchymal (VIM, N-CAD) and dysplasia (CA19-9, MUC5AC) marker expression together with moderate levels of proliferation (Ki-67). Such changes have been previously only found in* CDKN2A* deficient HUES8-derived PDLO grafts but not in wildtype grafts [13].

To further approach the complexity of a PDAC mutational landscape, we modeled the interplay between the specific *CDKN2A*^WT/#^ variant and the acquisition of the most important PDAC driver mutation *KRAS^G12D^* in PDLOs. We observed a lumen-filling phenotype after Dox-induced *KRAS^G12D^* expression in *CDKN2A*-mutated PDLOs in vitro. This change in morphology was reflected by loss of epithelial polarity, an increase of effectors of cell cycle arrest, and a partial initiation of an EMT program indicative for early neoplastic growth [39,40,41].

In agreement, *CDKN2A^WT/#^ KRAS^G12D^* xenografts developed into a high-grade precancer-like lesion and an invasive PDAC-like tumor. Of note, high *KRAS^G12D^* levels spatially correlated with disseminating cells acquiring a mesenchymal morphology. However, despite the heterozygous *CDKN2A* P16^WT/G101W^/P14^WT/R115L^ germline variant, cell cycle checkpoints were still activated upon *KRAS^G12D^* expression, posing a potentially relevant tumor progression barrier.

Both, P16/RB cell cycle inhibition and the effector P21 were activated, albeit we did not observe P14-mediated stabilization of P53 upstream of P21 induction. Hence, compensatory P21 induction independent of P53 appears likely. In addition, further analyses are required to investigate the P53-independent role of P14 for PDAC development from *CDKN2A^WT/#^* *KRAS^G12D^* PDLO grafts.

A homogenous nuclear and cytoplasmic localization of P16 has been previously shown to be important for proper growth restriction [42,43]. Several loss-of-function *CDKN2A* mutations including the here investigated P16-G101W variant caused a shift towards nuclear P16 expression and resulted in impaired CDK4/6 binding, enhanced CDK4/6 kinase activity, hyper-phosphorylation of RB, and therefore cell cycle progression [42,43,44,45,46]. In contrast, we observed a strong cytoplasmic expression of P16 in the highly dysplastic region of the *CDKN2A^WT/#^* *KRAS^G12D^* PDAC-like tumor resulting in tumor growth restriction suggesting partial compensation by the heterozygous *CDKN2A* WT allele upon high oncogenic burden. Indeed, expression of *KRAS^G12D^* in hESC-derived PDLOs with a homozygous ablation of *CDKN2A* caused the formation of highly de-differentiated PDACs in six out of six xenografts, which have completely overcome P16-RB and P14/P53/P21 cell cycle checkpoint control [13]. Hence, the frequency of tumor development, the need for additional driver mutations potentially including loss-of-heterozygosity, and subsequent progression into metastatic cancer of *CDKN2A^WT/#^* *KRAS^G12D^* PDLO xenografts needs to be determined in larger cohorts in future studies. Such approach should ideally include a repaired *CDKN2A^WT/WT^* *KRAS^G12D^* PDLO control to segregate the effect of the patient gene variant from potential genetic covariants.

## 4. Materials and Methods

### 4.1. Materials Availability

Transfer of the *piggyBac KRAS^G12D^* plasmid requires the permission of Knut Woltjen as original provider of the *piggyBac* construct and a completed Material Transfer Agreement. Generated iPSC lines from patients with *CDKN2A* variants as well as other materials can be provided from the lead contact (Meike.Hohwieler@uni-ulm.de) upon request and with a completed Material Transfer Agreement.

### 4.2. Patient Material

Keratinocytes of two individuals, a female (P1, IV-1) and a male (P2, IV-2), were reprogrammed in this study. This pair of siblings is included in a structured surveillance program in our outpatient clinic due to a complex family history of cancer. External genomic investigations by the “Medizinisch Genetisches Zentrum” (MGZ) Munich on the mother of the two patients (III-1) found neither a pathogenic BRCA1/2 point mutation by high resolution melting (HRM) and sequencing nor an exon deletion by multiplex-dependent probe amplification (MLPA). A subsequent screen in 94 oncogenes/tumor suppressor genes associated with hereditary cancer diseases identified the pathogenic NM_000077.4:c.301G>T variant encoded by the *CDKN2A/P16^INK4A^* locus (NP_000068.1:p.G101W). This variant corresponds to a NM_058195.3:c.344G>T variant in the *CDKN2A/P14^ARF^* transcript (NP_478102.2:p.Arg115Leu). No further pathogenic variant was found in the screen again including the *BRCA1/2* gene locus. Targeted Sanger sequencing reidentified the specific *CDKN2A* variant in the two patients and several further relatives. Patient 1 (IV-1) was additionally examined to exclude a potentially undetected BRCA1/2 mutation. Again, no pathogenic point mutations were found by sequencing (BRCA1) or denaturing high performance liquid chromatography (DHPLC) (BRCA2), and no exon deletion was found by MLPA.

Written informed consent of the two patients was given for material extraction and scientific use of reprogrammed cells. The study and reprogramming were approved by the local ethics committee at Ulm University (reference no. 68/11-UBB/bal.). Plucked patient hair was used to isolate keratinocytes and to establish iPSCs. Briefly, 6-well plates were precoated with Matrigel (Basement Membrane Matrix; Corning, Corning, NY, USA) (1:10 in EpiLife; Gibco, Thermo Fisher Scientific, Waltham, MA, USA) and hair roots were transferred in a drop of 1:5 diluted Matrigel in EpiLife. After 24 h, conditioned mouse embryonic fibroblast medium supplemented with 10 ng/mL FGF2 (R&D Systems, Minneapolis, MN, USA), and 10 µM ROCK inhibitor (Y-27632; Abcam, Cambridge, UK) was added and changed every four days. When keratinocytes grew out from the hair root, the medium was changed to EpiLife with human keratinocyte growth supplement (Gibco) and 10 µM ROCK inhibitor with media change every second day. For further cultivation, cells were split with TrypLE and seeded on Collagen IV-coated wells (20 µg/mL, Sigma-Aldrich, Merck, Darmstadt, Germany).

### 4.3. Reprogramming Strategy for iPSC Generation

Patient keratinocytes were reprogrammed to P16^G101W^/P14^R115L^ iPSCs by a lentiviral approach as previously described [18]. When keratinocytes reached a confluency of 75%, infection of around 200,000 cells with 1 × 10^8^ viral genome copies of hOKSM-dTomato virus [47] was performed on two subsequent days. Virus was diluted in growth medium containing 8 μg/mL polybrene (Sigma-Aldrich). Infected keratinocytes were harvested the next day using TrypLE and transferred in a 1:3 or 1:4 ratio to 6-wells with inactivated rat embryonic fibroblasts (REFs) as feeder layer. Gamma-irradiation with 30 Gy [20] for inactivation was carried out one day in advance and 3.5 × 10^8^ REFs were seeded per well. On the feeder layer, cells were cultivated in hiPSC medium composed of Knockout DMEM (Gibco), 20% knockout serum replacement (Gibco), 2 mM L-Glutamine (Gibco), 100 µM NEAA (Sigma-Aldrich), 100 µM β-Mercaptoethanol (Merck Millipore, Darmstadt, Germany), 1% Antibiotic-Antimycotic (Sigma), 50 µg/mL L-Ascorbic acid (A4544, Sigma-Aldrich), 10 ng/mL FGF2, and 10 µM ROCK inhibitor at 37 °C, 5% CO_2_, and 5% O_2_ with daily media change. iPSC colonies arising around 14 days later were transferred again onto inactivated REFs by manual picking. For further clonal expansion, feeder-free iPSC cultures were established by plating picked colonies on Matrigel-coated wells [20,48]. We verified the patients’ *CDKN2A* variant in keratinocytes and iPSCs by PCR amplification (CDKN2AiPSC-exon2-fwd, CCGCAGAAGTTCGGAGGATA;* CDKN2A*iPSC-exon2-rev, CTTTGGAAGCTCTCAGGGTACA) and sequencing (Eurofins Genomics, Ebersberg, Germany).

### 4.4. Embryonic and Induced Pluripotent Stem Cells

The applied human embryonic stem cell (hESC) line HUES8 has been obtained from Harvard University (RRID:CVCL_B207). hESCs and iPSCs were cultured in mTesR1 or mTesR Plus (STEMCELL Technologies, Vancouver, BC, Canada) at 5% CO_2_, 5% O_2_, and 37 °C on plates precoated with hESC-qualified Matrigel (Corning). Cells were split in a 1:4 to 1:6 ratio twice a week. After washing with PBS, the cells were incubated with TrypLE (Gibco) for 5 min at 37 °C. Detached cells were flushed with DMEM-F12+GlutaMAX (Gibco), transferred to a collection tube, and centrifuged at 200× *g* for 5 min. After resuspension in mTesR medium containing 10 µM ROCK inhibitor, cells were seeded again on Matrigel-coated culture plates.

### 4.5. All-In-One piggyBac-System and Nucleofection

A *piggyBac* (PB) transposon system was applied to generate pluripotent stem cells overexpressing *KRAS^G12D^* in a Dox-dependent manner. Therefore, the All-in-One PB vector established by Kim et al. [19] was modified through gateway cloning of mutated *KRAS*. The respective cDNA sequence was amplified by PCR from the pBabe-KRAS G12D plasmid (Addgene plasmid #58902, a gift from Channing Der). First, *KRAS^G12D^*-specific primers (attB1-SpeI-HindIII-(N-HA)KRAS_G12D-fwd: aaaaagcaggcttcactagtgtctttcataagcttatgtatccatatgatgtgcccgact; attB2-KRAS_G12D-rev: agaaagctgggtgtgacacacattccacagggt) were used followed by a second PCR step with primers harboring *attB1/attB2* adapter sites required for gateway cloning (attB1-adapter-primer: ggggacaagtttgtacaaaaaagcaggct; attB2-adapter-primer: ggggaccactttgtacaagaaagctgggt) [49]. Purification of the PCR product was performed with the Wizard SV Gel and PCR Clean-Up System (Promega, Madison, WI, USA) according to manufacturer’s recommendations. Amplified gene sequences were integrated into the pDONR201 vector (Thermo Fisher Scientific, Waltham, MA, USA) using gateway cloning as described by Gloeckner et al. [49] and the BP Clonase II enzyme mix (Thermo). Final integration into the Destination vector PB-TAC-ERP2 (Addgene plasmid #80478, a gift from Knut Woltjen [19]) was performed by a second reaction with the LR Clonase II enzyme mix (Thermo). The final PB-TAC-ERP2-(N-HA)KRAS_G12D (short: PB-KRAS) plasmid sequence was validated by Sanger sequencing (PB-seq-fwd: agctcgtttagtgaaccgtcagatc; PB-seq-rev sequencing: gtacaagaaagctgggt). *CDKN2A*-mutated iPSCs were co-transfected with PB-KRAS and the transposase plasmid (SBI Biosciences #PB200PA-1 [50]), at a ratio of 3.3:1. For nucleofection, the P3 primary cell 4D Nucleofector X Kit S and a 4D Nucleofector (both Lonza, Basel, Switzerland) were used at a CA-137 pulse setting. After transfection of 200,000 cells with 0.5–1 µg of DNA (in total), mTesR1 medium supplemented with 10 µM ROCK inhibitor was added to the cells. After 3–5 min at 37 °C, cells were transferred to Matrigel-precoated 96-wells. Selection of cells that stably integrated the PB expression cassette into the genome was started 24 h post nucleofection using 1 µg/mL puromycin (Sigma-Aldrich). Karyotyping of the P16^G101W^/P14^R115L^ *KRAS^G12D^* iPSC lines was performed by the Department for Human Genetics at Ulm University according to routine clinical guidelines. At least 20 metaphases were analyzed for one clonal and one bulk cell line, and the set of chromosomes was inconspicuous.

### 4.6. Pancreatic Progenitor Differentiation

To induce the differentiation of hPSCs to PPs, an optimized protocol recently published by our group [13] and based on two previous publications [18,51] was applied. Briefly, 300,000 cells in mTesR medium with 10 µM ROCK inhibitor were seeded per 24-well coated with 0.5 mg/mL GFR-Matrigel (Corning). Differentiation was started on the next day when cells were 90% confluent by adding d0 medium. Media composition of BE1 and BE3 basal media as well as detailed descriptions of added cytokines throughout the 13-days differentiation protocol are described by Breunig et al. [13]. Cells were incubated at 37 °C with 5% CO_2_ with daily medium change. Flow cytometry analysis was applied for quality control at specific stages. At the definitive endoderm (DE; d3) stage, at least 95% had to be CXCR4/cKIT double positive and at pancreatic progenitor stage (PP; d13), the threshold was set to 60% PDX1/NKX6-1 double positive cells to further differentiate PPs into PDLOs.

### 4.7. PDLO Culture

For the differentiation towards pancreatic duct-like organoids (PDLOs), PPs were transferred to well-plates coated with undiluted GFR-Matrigel (in a 3D culture format based on Xiang and Muthuswamy [52]). At day 13, cells were detached using TrypLE and washed with BE3 medium. Per each 12-well, 100,000 cells were seeded in phase I differentiation media supplemented with 5% GFR. Ductal differentiation media for phase I (d13–d19) and phase II (from d20) were described by Breunig et al. [13]. Medium was changed twice a week with 5% GFR-Matrigel being directly added to the cold medium before use.

To passage PDLOs, organoids were recovered from the Matrigel with 1 mg/mL Collagenase/Dispase (Roche, Rotkreuz, Switzerland) diluted in DMEM-F12+GlutaMAX. After incubation for 2–4 h at 37 °C, PDLOs were resuspended in cold neutralization solution (1% BSA, 1% P/S in DMEM), centrifuged (200× *g*, 5 min), and washed with PBS. A single cell suspension was generated by incubation with Accutase (Sigma-Aldrich) for 30 min at 37 °C in a water bath and intermittent pipetting up and down after 15 min and 30 min. After stopping the reaction by adding neutralization solution, cell pellets were resuspended in phase II medium supplemented with 10 µM ROCK inhibitor and were seeded on Matrigel-coated plates. For transgene induction after PDLO passaging, 3 µg/mL Doxycycline was added to the culture medium.

### 4.8. Orthotopic Transplantation of PDLOs

Orthotopic transplantation of human PDLOs was performed in NOD *scid* gamma (NSG) mice (NOD.Cg-Prkdc^scid^ Il2rgtm1Wjl/SzJ strain; Charles River; RRID:BCBC_4142) at an age between 6 and 12 weeks and mice were kept in a hygiene barrier room to reduce pathogenic burden.

Prior to transplantation, day 27 or day 41 PDLOs were dissociated to single cells as detailed above, resuspended in phase II PDLO medium containing 20 µM Y-27632 (final concentration was 10 µM), mixed with GFR-Matrigel at a 1:1 ratio, and stored on ice. NSG mice that were treated with the analgesic Tramadol (Grünenthal, Aachen, Germany) three days in advance were anesthetized by isoflurane inhalation and s. c. injection of 1 mg/kg meloxicam 30 min before surgery. After disinfection, the skin and the peritoneum were opened at the site of the spleen and pancreas. Then, 0.5–1 × 10^6^ PDLO cells were injected in 40–50 µL 50% Matrigel/50% medium solution into the pancreatic tail. While the peritoneum was sewn up with 5-0 polyglactin coated vicryl suture (Ethicon, Johnson & Johnson, Neuss, Germany), the skin was closed with surgical staples. Tramadol treatment was maintained for one week. After one week, also the staples were removed. For in vivo oncogene induction, the drinking water was supplemented with Doxycycline in a final concentration of 400 µg/mL and 5% sucrose starting one day after transplantation. After eight weeks, mice were sacrificed and pancreata were fixed in 4% PFA solution and paraffin embedded for further analysis.

### 4.9. qPCR Experiments

PDLOs were recovered from the Matrigel with 1 mg/mL Collagenase/Dispase solution for around 2 h as described in the PDLO culture section. After recovery and washing with PBS, cells were lysed. RNA was isolated using the GeneJET RNA Purification Kit (Thermo), transcribed to cDNA with the iScript cDNA Synthesis Kit (Bio-Rad, Hercules, CA, USA), and qPCR measurements were performed on a Rotor Gene Q cycler (Qiagen, Hilden, Germany) according to manufacturer’s instructions. Self-designed primers were applied at a concentration of 0.25 µM, and the sequences are listed together with commercially acquired primers in Table 1.

### 4.10. Flow Cytometry

Whereas organoids were processed to single cells with Collagenase/Dispase and Accutase (described in the PDLO section), monolayer cells (DE, PE, PP cells) were harvested using TrypLE. For intracellular staining, cells were washed with PBS before fixation in 4% PFA, diluted in PBS with 100 mM sucrose (both Sigma-Aldrich), for 25 min on ice. After two washing steps with PBS (1000× *g*, 5 min), cells were blocked for 30 min on ice using 5% normal donkey serum (Jackson ImmunoResearch, West Grove, PA, USA) diluted in 0.1% Triton X-100/PBS (PBS-T) as a blocking solution. After centrifugation at 3000× *g* for 5 min, primary antibodies [PDX1 (R&D, AF2419, 1:500), NKX6-1 (DSHB, Iowa City, IA, USA, F55A12, 1:150), P21 (Abcam, ab109520, 1:100), Ki-67 (Thermo, MA5-14520, 1:1000), and HA-tag (Cell Signaling, Danvers, MA, USA, 3724, 1:1600)], diluted in the blocking solution, were added to the cells for o/n incubation at 4 °C. In case of directly fluorophore-labeled antibodies [PDX1-PE (BD, Franklin Lakes, NJ, USA, 562161, 1:35), NKX6-1-APC (BD, 563338, 1:35)], cells were incubated with the antibody solution for 90 min on ice. After incubation with primary antibodies, cells were washed twice with 2% normal donkey serum in 0.1% PBS-T (wash solution). In case primary antibodies were not directly fluorophore-labeled, cells were additionally incubated with secondary antibodies (Alexa Fluor, Thermo), diluted 1:500 in the blocking solution, and incubated with Alexa Fluor secondary antibodies (Thermo) for 90 min on ice. After two washing steps, cells were resuspended in washing solution, filtered, and measured using an LSR II flow cytometer (BD).

Additionally, CXCR4-PE (Thermo, MHCXCR404, 1:50) and cKIT-APC (Thermo, CD11705, 1:100) surface staining on DE cells was performed on living cells without fixation of the cells and the detailed protocol can be found in Breunig et al. [13].

### 4.11. Cell Cycle Analysis

PDLO cultures were incubated with 10 µM EdU for 4 h prior to recovery and singularization of PDLOs. Cell cycle phases of PDLO cells were analyzed by subsequent staining with the Click-iT EdU Alexa Fluor 647 Assay Kit (Life Technologies, Thermo) followed by flow cytometry measurement. Further experimental details are described in [13].

### 4.12. ICC Staining

The hESCs and PPs were stained by immunocytochemistry (ICC/IF). For improved staining quality, hESCs were cultured and differentiated on Matrigel-coated 24-well ibiTreat µ-plates (IBIDI, Gräfelfing, Germany). For fixation, cells were washed with PBS and incubated in 4% PFA+100 mM sucrose solution for 20 min at RT. After three rounds of PBS washing, 50 mM NH_4_Cl (Sigma-Aldrich) was added for quenching for 10 min again followed by three washing steps. Cells were then permeabilized using PBS-T, blocked for 45 min in 5% normal goat serum (Jackson ImmunoResearch) or 5% DS in PBS-T and incubated o/n at 4 °C with primary antibodies diluted in blocking solution. Next, cells were washed three times with PBS before secondary antibody solution was added for 1 h at RT in the dark. After PBS wash, cells were incubated with 500 ng/mL DAPI in PBS for 10 min and finally kept in PBS for imaging. As primary antibodies OCT4 (Santa Cruz Biotechnology, Dallas, TX, USA, sc-5279, 1:200), NANOG (Cell Signaling, 3580, 1:500), SSEA4 (Cell Signaling, 4755, 1:500), PDX1 (R&D, AF2419, 1:500), NKX6-1 (DSHB, F55A12, 1:150), and as secondary antibodies Alexa-Fluor conjugated antibodies (1:500) were used at specified concentrations. SSEA4 staining was performed without permeabilization step and plates were imaged on a BZ-9000 fluorescence microscope (Keyence, Osaka, Japan).

### 4.13. Paraffin Embedding of PDLOs

PDLOs were directly fixed in Matrigel wells. After washing with PBS, the cultures were incubated with 4% PFA+100 mM sucrose o/n at 4 °C. After removal of the fixation solution and two PBS washes, PDLOs were collected in a reaction tube and spun down at 3000× *g* for 3 min. Pre-embedding in 2% agarose (Sigma) was followed by histological processing including serial dehydration, paraffin embedding, and sectioning to 4-µm tissue slices using SuperFrost Ultra Plus microscope slides (Thermo).

### 4.14. Staining on Paraffin Tissue Sections

Formalin-fixed paraffin-embedded (FFPE) tissue and PDLO sections were either stained with Hematoxylin and Eosin (H&E) according to standard protocols or by immunofluorescence (IF) and immunohistochemistry (IHC). IF and IHC staining required rehydration in an ethanol series before antigen retrieval (see Table 2). Heat-mediated or enzymatic antigen retrieval was performed as detailed in Breunig et al. [13]. After antigen retrieval, IHC slides were incubated with the primary antibody solution in a wet chamber for 30 min at RT. After washing with 0.5% Triton X-100/PBS (PBS-T), antibody detection was performed with the Dako Detection Kit (ABC, Dako Agilent, Santa Clara, CA, USA). For nuclear counterstaining, 20% Hematoxylin solution (Merck) was added for 30 s. After rinsing with tap water for 5 min, slides were mounted with Aquatex (Merck).

In case of IF, antigen retrieval was followed by a permeabilization step with PBS-T for 30 min at RT and by two washing steps with PBS. Primary antibodies were added for staining o/n at 4 °C. After three PBS-T washes (5 min each), sections were stained with secondary antibodies (Alexa Fluor, 1:500) for 90 min at RT in the dark. 500 ng/mL DAPI was added during this incubation for visualization of nuclei. Slides were then washed three times with PBS-T, once with dH_2_O, and finally mounted with Fluoromount-G (SouthernBiotech, Birmingham, AL, USA). All antibodies were applied in Antibody Diluent (Zytomed Systems, Berlin, Germany).

### 4.15. Statistical Analysis

Statistical analysis was performed using the GraphPad Prism 8 software (GraphPad Software, San Diego, CA, USA) and the applied tests are specified in the figure legends. Statistical significance was defined as follows: * *p*-value < 0.05, ** *p*-value < 0.01, *** *p*-value < 0.001, **** *p*-value < 0.0001; only significant comparisons are denoted in the plots.

#### 4.15.1. Flow Cytometry and qPCR of PDLOs

For statistical analysis, data obtained from three different cell lines (two bulk and one monoclonal) were incorporated. Each cell line was considered as independent experiment.

#### 4.15.2. Bright Field Image Analysis of PDLOs

Bright field images and IF images of the mCherry reporter signal were taken from PDLO cultures at the end point (7 or 9 days after Dox treatment). To quantify the number of lumen-filled organoids, a semi-automatic image analysis was conducted using an algorithm in the Fiji software [53].

#### 4.15.3. Quantification of IF images

Analysis of IF images was performed either manually or automatically using Fiji [53]. In case of manual analyses, the operator was blinded. The number of γH2AX positive cells was determined by an automated algorithm using Fiji and γH2AX expression was normalized to the number of DAPI positive cells.

To assess PDLO polarity, organoids with at least 50% of cells showing a distinct intercellular expression of CLDN1 were categorized into CLDN1 positive (intact epithelial polarization). Similarly, organoids were classified into organoids with a clear luminal expression of ZO-1 and into organoids presumably lacking apico-basal polarity. To assess EMT, organoids were manually classified into N-CAD positive organoids, when at least one cell expressed N-CAD, and into VIM^high^ organoids, when a strong VIM intensity was observed in several cells within one organoid. For statistical analysis, two-sided Fisher’s exact tests were performed based on contingency tables compiled from the number of positive and negative cells/organoids. The number of quantified cells/organoids was comparable between each cell line and each condition and IF images from two independent cell lines were incorporated.

## 5. Conclusions

PDLOs from patient iPSCs represent an experimental model to study individualized tumor development in the pancreas. In contrast to primary human PDAC-derived organoids, this model starts from a specific genetic background and will thereby be suitable for screening drugs within the earliest time frame of tumorigenesis. Upon repair of gene variants in patient-specific iPSCs, the impact of distinct predisposing variants can be dissected in the future. Moreover, stage-specific oncogene expression and whole exome profiling in long-term experiments will also provide new insights into pathophysiological mechanisms of distinct routes of pancreatic tumor progression.

## Figures and Tables

**Table 1:** qPCR primers used in this study.

Gene	Self-Designed Primer Fwd	Self-Designed Primer Rev	Quantitect
FN1	-	-	QT00038024
HMBS	-	-	QT00494130
N-CAD	-	-	QT00063196
P14	ttttcgtggttcacatcccg	gggcgctgcccatcat	-
P21	gcgccatgtcagaaccgcct	gcaggcttcctgtgggcgga	-
SLUG	cagtgattatttccccgtatc	ccccaaagatgaggagtatc	-
SNAIL	gctccttcgtccttctcctc	tgacatctgagtgggtctgg	-
VIM	gacaatgcgtctctggcacgtctt	tcctccgcctcctgcaggttctt	-

**Table 2:** IHC/IF staining conditions for antibodies used in this study.

Antibody	Species	Company	Catalogue No.	Condition	Dilution
CA19-9	mouse	Thermo	MA5-12421	ST Citrate	1:500
CLDN1	rabbit	Abcam	ab15098	ST Tris	1:100
cl-CASP3	rabbit	Cell Signaling	9664	ST Citrate	1:1000
E-CAD	mouse	BD Bioscience	610182	ST Citrate	1:1000
HA-tag	rabbit	Cell Signaling	3724	ST Citrate	1:500
Ki-67	mouse	Dako	M7240	ST Citrate	1:200
Ki-67	rabbit	Invitrogen	MA5-14520	ST Citrate	1:100
KRT19 (IF)	mouse	Dako	M0888	ST Citrate	1:100
KRT19 (IHC)	mouse	Dako	M0888	Pronase	1:100
KRT7	mouse	Dako	M7018	Pronase	1:200
mCherry	rabbit	Abcam	ab167453	No AGR	1:500
MUC5AC	mouse	Santa Cruz	sc-33667	ST Citrate	1:100
N-CAD (IF)	rabbit	Cell Signaling	13116	ST Citrate	1:100
N-CAD (IHC)	rabbit	Cell Signaling	13116	ST Tris	1:100
P14	mouse	Cell Signaling	2407S	ST Citrate	1:50
P16	rabbit	Abcam	ab108349	ST Citrate	1:400
P21	rabbit	Abcam	ab109520	ST Citrate	1:300
P53	mouse	Santa Cruz	sc-47698	ST Citrate	1:100
pRB	rabbit	Cell Signaling	8516	ST Citrate	1:200
RB	mouse	Cell Signaling	9309	ST Citrate	1:400
VIM	rabbit	Cell Signaling	5741S	ST Citrate	1:500
ZO-1	mouse	Thermo	33-9100	ST Citrate	1:500
γH2AX	rabbit	Cell Signaling	9718	ST Citrate	1:400

AGR, antigen retrieval; ST, steamer.

## Data Availability

The data presented in this study are contained within the article and further source data are available from the corresponding author upon request. This study did not generate datasets or new codes to be deposited.
